# Supplementary material for: Indigenous Australians’ Experiences of Cancer Care: A Narrative Literature Review
Source: Int J Environ Res Public Health. 2022 Dec 16;19(24):16947. doi: 10.3390/ijerph192416947 (PMC9779788; doi:10.3390/ijerph192416947)
Supplement: Supplementary file 1 [file ijerph-19-16947-s001.zip › ijerph-2063110-supplementary.pdf]

Table S1. Databases search results.

	Query	Results
Pubmed	((((((((australia[mh] OR australia*[tiab]) AND (oceanic ancestry group[mh] OR aborigin*[tiab] OR indigenous[tw])) OR (torres strait* islander*[tiab])) AND medline[sb]) OR ((((au[ad] OR australia*[ad] OR australia*[tiab] OR northern territory[tiab] OR northern territory[ad] OR tasmania[tiab] OR tasmania[ad] OR new south wales[tiab] OR new south wales[ad] OR victoria[tiab] OR victoria[ad] OR queensland[tiab] OR queensland[ad]) AND (aborigin*[tiab] OR indigenous[tiab])) OR (torres strait* islander*[tiab])) NOT medline[sb]) AND English[la])) AND ((((((australia[mh] OR australia*[tiab]) AND "oceanic ancestry group" OR "Native Hawaiian or Other Pacific Islander"[Mesh] OR aborigin*[tiab] OR indigenous[tw])) OR (torres strait* islander*[tiab])) AND medline[sb]) OR ((((au[ad] OR australia*[ad] OR australia*[tiab] OR northern territory[tiab] OR northern territory[ad] OR tasmania[tiab] OR tasmania[ad] OR new south wales[tiab] OR new south wales[ad] OR victoria[tiab] OR victoria[ad] OR queensland[tiab] OR queensland[ad]) AND (aborigin*[tiab] OR indigenous[tiab])) OR (torres strait* islander*[tiab])) NOT medline[sb]) AND English[la])) AND (cancer)) AND (((((healthcare) OR (Healthcare service)) OR (community)) OR (health service)) OR (healthcare provider))	494
CINAHL	(aboriginal and torres strait islander or indigenous australians) OR (indigenous or native or aboriginal or indians or first nations) AND Australia AND Cancer AND (healthcare OR healthcare systems OR health services OR community)	164
Scopus	(TITLE-ABS-KEY (aboriginal AND torres and strait AND islander OR indigenous)) AND (TITLE-ABS-KEY (Cancer)) AND (ALL (health AND care OR health AND care AND service OR Health AND service OR community)	110

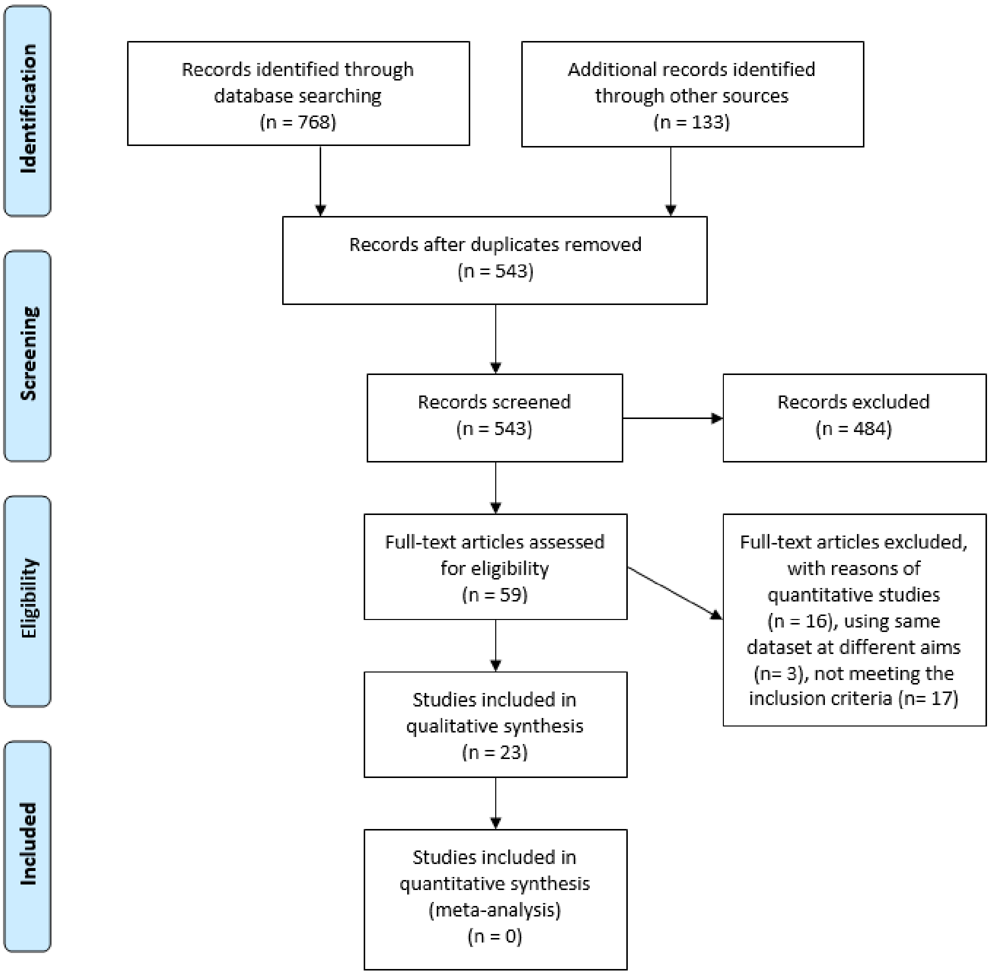

Figure S1. PRISMA flow diagram of study inclusion.
